# Demand for family planning satisfied by modern methods in Ghana: trends and inequalities (2013–2022)

**DOI:** 10.1186/s12889-025-22022-w

**Published:** 2025-05-01

**Authors:** Akua Amponsaa Obeng, Cauane Blumenberg, Seth Kwaku Afagbedzi, Yohannes Dibaba Wado, Kristen Nilsen

**Affiliations:** 1 Department of Biostatistics, School of Public Health, University of Ghana, Legon-Accra, Ghana; 2 International Centre for Equity in Health, Federal University of Pelotas, Pelotas, RS Brazil; 3 Causale Consultoria, Pelotas, RS Brazil; 4 African Population and Health Research Center, Nairobi, Kenya; 5 WorldPop, School of Geography, University of Southampton, Highfield Campus, Southampton, SO17BJ UK; 6 Department of Social Statistics and Demography, University of Southampton, Southampton, UK

**Keywords:** Family planning, Contraceptive, Modern contraceptive, Ghana

## Abstract

**Background:**

In Ghana, while coverage of demand for family planning satisfied by modern methods (mDFPS) has increased, substantial inequalities persist across demographic and geographic factors. This study aims to assess mDFPS trends from 2013 to 2022, with a focus on inequalities related to residence, education, and wealth, and identifying the determinants of mDFPS.

**Methods:**

Using data from Performance Monitoring Action (PMA) (2013–2017) and Ghana Demographic and Health Survey (DHS) (2022), an evaluation of the trends of demand for family planning satisfied by modern methods (mDFPS) was assessed from 2013 to 2022 with the corresponding annual average rate of change. Absolute complex measures of inequalities (SII and WMADM) were used to identify wealth, education and regional related inequalities in mDFPS coverage. A binary logistic regression was used to assess factors influencing mDFPS.

**Findings:**

The coverage of women with a demand for family planning satisfied with modern methods increased from 33.0% to 49.5% between 2013 to 2022. An overall 3.8% annual increase in mDFPS was observed from 2013 to 2022. A decreasing trend in wealth, education and regional inequalities were observed over the years. However, women with no education and those from the Northern region of Ghana have consistently had the lowest mDFPS coverage over the years, and they continue to lag. Women aged 20–35 have a 28% increase in odds [95%CI:1.01–1.63; *p *= 0.038] of family planning satisfaction by modern methods compared to those aged 15–19. Mothers currently working have a 27% increased odds of family planning satisfaction by modern methods compared to those who are not working [95%CI: 1.07–1.51; *P* = 0.007]. Furthermore, women who are married or co-habiting have a 33% decrease in odds of family planning satisfaction by modern methods compared to those who are single [95%CI: 0.56–0.84; *P* < 0.001].

**Conclusion:**

Reductions in educational and wealth-related inequalities in mDFPS coverage have been observed over time; however, persistent challenges emphasize the need for targeted interventions. Prioritizing equitable access for poorer, less educated women and addressing regional disparities, particularly in the Northern region of Ghana, are crucial to achieving inclusive family planning services.

**Supplementary Information:**

The online version contains supplementary material available at 10.1186/s12889-025-22022-w.

## Background

Modern family planning methods are important public health interventions worldwide, enabling individuals, particularly women, to exercise control over their reproductive health and make informed decisions regarding the timing and spacing of their children [1]. These methods including oral contraceptives, IUDs, implants, male and female condoms contribute significantly to achieving global development goals, such as the United Nations' Sustainable Development Goals (SDGs) 3 and 5, which focus on ensuring good health and well-being and achieving gender equality [2, 3]. Evidence shows that their widespread use can dramatically reduce maternal and child mortality rates, with the potential to prevent up to 70% of maternal deaths, 58% of under-five deaths, and decrease unintended pregnancies by 40% [4]. In recognition of these substantial benefits, the international community and various national governments have implemented policies and invested resources to improve access to modern family planning services. These policies include the FP2020 Initiative, and the Maputo Protocol, resulting in notable increases in modern contraceptive utilization worldwide [2, 5].

Despite global advancements in contraceptive prevalence, a significant gap remains in access to family planning services. More than 200 million women in low- and middle-income countries who wish to avoid pregnancy are still not using modern contraceptive methods, highlighting a major public health challenge [6–9]. To meet global targets such as achieving 75% coverage of demand for family planning satisfied by modern methods (mDFPS), they must increase by an estimated 2.2 percentage points annually from 2014 to 2030 across 63 nations. However, only nine of these countries are projected to meet this benchmark at the current rates of progress [10]. The lack of access to modern family planning increases the risk of unintended pregnancies, leading to unsafe abortions and heightened maternal mortality and morbidity.

On a regional level, areas such as South Asia, Latin America, and the Caribbean have made notable progress, with mDFPS coverage reaching approximately 70% [2]. In contrast, West and Central Africa continue to struggle with significantly lower coverage rates, averaging only 33%. Within these regions, individual country rates vary between 17.6% and 51.5%, further emphasizing the disparities in access to modern contraceptive methods [3]. The lower coverage has been attributed to the opposition from partners, concerns about contraceptive side effects, and a low perceived risk of pregnancy due to infrequent sexual activity [11–13]. Unlike common assumptions, lack of access to contraceptives is not a found to be a predominant barrier [14]. Cultural pressures, such as expectations for early pregnancies after marriage and the value placed on large families for labor, particularly in rural areas, further hinder modern contraceptive use [11, 13]. These factors have resulted in persistently high fertility rates and low levels of female empowerment, which have further exacerbated these challenges and constrained progress in improving reproductive health outcomes in these areas [15–17].

In Ghana, mDFPS coverage among married women of reproductive age in Ghana increased by 28% between 1993 and 2022 while experiencing a sharp decline in fertility rate before stabilizing at 3.9 children per woman between 2017 and 2022 [13]. Concurrently, mDFPS coverage rose from 46% in 2017 to 47% in 2022, reflecting sustained efforts to expand access to modern contraceptives at the national level [18, 19]. However, significant inequalities persist across demographic factors such as age, education, wealth, and geographic location, with marked disparities between rural and urban areas [20].

Despite the progress made in Ghana, many gaps remain in understanding the evolution of mDFPS disparities in Ghana since 2014. A recent study relied on Demographic and Health Survey (DHS) data only up to 2014, leaving the trends over the past decade unexplored [16]. This study aims to bridge that gap by providing a comprehensive analysis of mDFPS from 2017 to 2022, focusing on inequalities by residence, age, education, and wealth, and investigating geographical disparities at the sub-national level and also, identifying the determinants influencing the uptake of modern family planning methods. This study will offer crucial insights for informed policy-making and the design of targeted interventions to enhance equitable access to family planning services.

## Methods

### Theoretical framework

The Social determinants of health (SDH) is the framework for this study and has been applied in similar studies [13, 21]. The Social Determinants of Health (SDH) framework explores how social, economic, and environmental determinants that influence health outcomes, including access to modern family planning methods [22]. These determinants create structural barriers or enablers that impact individuals' ability to achieve optimal health [22]. The SDH framework is particularly relevant for analyzing trends in the demand for family planning satisfied by modern methods (mDFPS) from 2017 to 2022, with a focus on inequalities related to residence, education, and wealth. For instance, women from rural areas often face limited healthcare infrastructure, while those with lower educational attainment may lack awareness or empowerment to utilize family planning services [23]. Similarly, economic disparities restrict access for poorer women, exacerbating reproductive health inequities [24]. This study highlights the interconnected social and economic factors driving disparities in mDFPS coverage, offering critical insights into how these systemic barriers can be addressed to enhance equitable access to family planning services. Thus, providing a comprehensive understanding of the root causes of inequality, enabling the development of targeted interventions to improve reproductive health outcomes.

### Study design and data source

The study design was a secondary data analysis using cross-sectional surveys from Ghana Performance Action Data (PMA) and Ghana Demographic and Health Survey (DHS). A series of datasets from surveys between the period 2013–2017 (PMA) and 2022 (DHS) were utilised in this study for trend analyses. The PMA and DHS data are nationally representative cross-sectional surveys conducted in low- and middle-income countries [18, 25]. They utilize similar two-stage cluster sampling methods to select households and individuals for inclusion in the survey. Additionally, both surveys target women of reproductive age (usually 15 to 49 years old) and collect information on a wide range of health-related topics, including maternal and newborn health, family planning practices, nutrition, and access to healthcare services. They are considered highly comparable and are representative at national, regional, and sometimes even sub-national levels [18, 25]. This study was restricted to all women of reproductive age (15–49 years).

The DHS and PMA datasets have a high response rate, exceeding 95%. Given this high level of participation, this study assumed that any missing data is likely missing completely at random. This suggests that there are no systematic differences in the observed characteristics between individuals with missing data and those with complete data, thus minimizing bias in the results.

### Dependent variable

The dependent variable is the demand for family planning satisfied by modern methods. (mDFPS). This indicator is defined as the proportion of women between the ages of 15 and 49 who are presently using modern methods of contraception and who want to either delay or avoid having children and was calculated using the current DHS method [26]. The numerator comprises all women aged 15–49 years using modern contraceptives. Male and female condoms, injectables, oral contraceptive pills, intrauterine devices (IUDs), implanted procedures, lactational amenorrhoea method (LAM), emergency contraception, and other contemporary methods not previously mentioned separately (such as vaginal rings or patches) are examples of modern methods of contraception. On the other hand, the denominator comprises of all women 15–49 years with both a met and unmet need (women with a demand for family planning).

### Inequality dimensions

The variables used as dimensions to assess inequality patterns are wealth, educational level and region of residence of all women with a demand for family planning. Wealth status was assessed using a wealth index, which was constructed based on household asset ownership and access to amenities. This index typically includes items such as ownership of durable goods, housing characteristics, access to utilities and other socioeconomic indicators. The wealth index was then divided into tertiles; Poor, Middle, and Rich wealth categories. Educational level was assessed categorically: No formal education, Primary/Junior (JSS) education, Secondary and Higher education. The region of residence was assessed categorically. This study utilized the 10 administrative regions of Ghana, instead of the current 16-region division. This choice ensured that data collected across different periods could be compared consistently, as earlier surveys and datasets were organized according to the 10-region structure.

### Covariates

Other variables included in the study as covariates to identify factors associated with mDFPS were age of mother, the number of living children, type of residence (Urban/Rural). The number of living children was treated as a continuous variable, reflecting the exact number of children each respondent had. Type of residence was categorized as either urban or rural to assess geographical disparities in access to family planning services. The age of the mother was categorised into three groups; 15–19, 20–35, 36–49.

### Data analysis

A trend analysis was conducted to analyse the trend in mDFPs from 2013–2022 using Stata 18 software. In this study, key variables for each year were aligned across surveys to ensure consistent definitions and coding. Sampling differences were adjusted using survey weights from both PMA and DHS. This approach enabled a year-by-year analysis of trends and patterns. To determine the Average Annual Rate of Change (AARC) in the coverage of demand for family planning satisfied by modern methods in percentages, the indicator estimates underwent a logarithmic transformation and were then regressed against the survey years using ordinary least-squares (OLS) models. Subsequently, the AARC was calculated using the formula AARC = (1—e^β) × −1. The estimates were weighted according to the standard error of prevalence at each time point to adjust for the differing time intervals between consecutive surveys and ensuring that time points with more precise prevalence estimates have a greater influence on the overall analysis, reducing the impact of less reliable estimates.

Absolute measures of inequalities were employed to assess inequality measures of mDFPs among women. To determine absolute inequalities in the coverage of demand for family planning satisfied by modern methods, the Slope index of inequality (SII) through logistic regression were computed. The SII measures the absolute difference in the prevalence of interest between ordered groups, expressed in percentage points [27]. SII was calculated based on wealth, indicating the absolute difference in coverage between the wealthiest and poorest women. Furthermore, SII was calculated based on educational level, indicating the absolute difference in coverage between women with higher education and those with no education. The Weighted Mean Absolute Difference to the Mean (WMADM) was used to calculate inequalities of coverage of mDFPS across regions. WMADM calculates the mean of the absolute differences between each subnational unit and the national average, considering only positive values and is weighted by population size in each region. When these measures (SII and WMADM) yield a result of 0, it indicates the absence of inequalities and greater absolute values imply greater degrees of inequality. The inequalities are further visualised using equiplots and bar plots [27].

A regional distribution of mDFPS for the years in Ghana was produced using the QGIS Software and visualized as maps respectively. The process involved obtaining a shapefile of Ghana’s regions and coverage of mDFPS data, then loading both into QGIS. A graduated symbology was applied to visually represent variations in mDFPS coverage across regions. A colour gradient from yellow to deep green was used, indicating a lower to higher coverage.

A binary logistic regression was also carried out to determine the determinants of mDFPS using the 2022 DHS data. The model accounted for weighting, stratification and clustering. The odds ratio and their corresponding confidence interval and P-values were presented. The fitness of the models was first assessed using the Macdan Rsquared and the overall probability of the model.

## Results

### Demographic characteristics of participants

The demographic characteristics of all women included in the study across different years from 2013 to 2022 are presented in Table S1 (Additional File 1). A total of 39,904 women of reproductive age were analysed in the study. The proportion of women aged 15–19 ranged from 17.9% to 20.1%, while those aged 36–49 saw a gradual increase from 26.7% in 2013 to 31.0% in 2022. Educational levels improved a consistent decline in women with no formal education (from 21.6% in 2013 to 15.5% in 2022) and a marked rise in those attaining senior high school education (16.6% to 60.5%). Urban residence saw a general increase, rising from 51.1% in 2013 to 57.0% in 2022. The proportion of women in the poor category ranged from 33.3% in 2013 to 33.4% in 2022, while the middle category ranged from 33.2% to 33.4%, and the rich category similarly ranged from 33.2% to 33.4%

Regionally, Ashanti consistently had the highest representation, ranging from 16.6% in 2016 to 20.7% in 2014 and stabilizing at 19.5% in 2022. Greater Accra followed closely, with proportions ranging from 15.5% in 2022 to 19.5% in 2017. The Northern region's representation fluctuated between 8.9% in 2015 and 11.7% in 2022. Regions with consistently lower representation included Upper East (3.3% to 6.9%) and Upper West (2.6% to 4.0%). Modern contraceptive use demonstrated an upward trend, increasing from 14.1% in 2013 to 23.4% in 2022.

### Trends in demand for family planning satisfied by modern methods

**Fig. 1:**
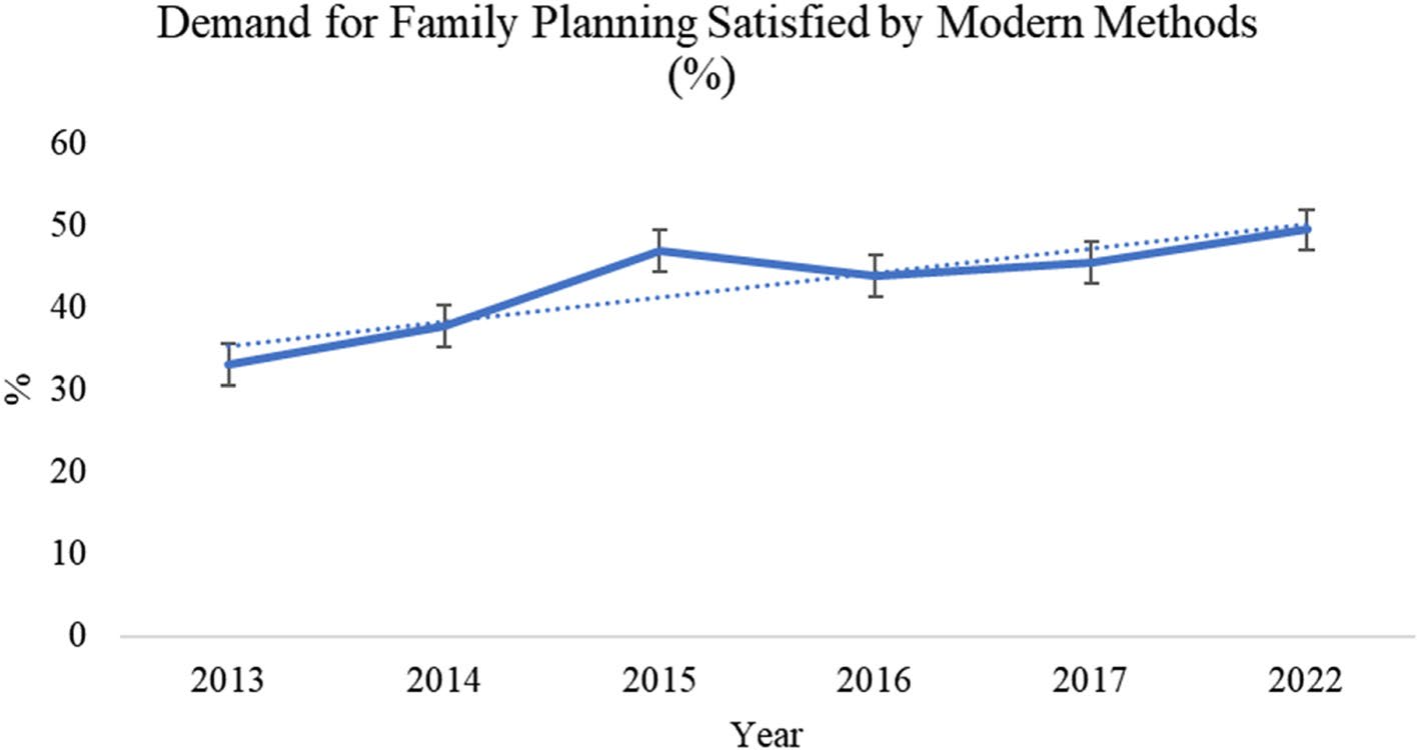
Trends in mDFPS from 2013 to 2022

The coverage of women with a demand for family planning satisfied with modern methods increased from 33.0% to 49.5% between 2013 to 2022 (Fig. 1). An overall 3.8% [95%CI; 3.3–4.0] increase per year in mDFPS was observed from 2013 to 2022.

Table S2 (See Additional File 1) presents trends in the demand for family planning satisfied by modern methods (mDFPS) between 2013 and 2022, disaggregated by key socio-demographic factors including; age, educational level, wealth, and residence. An upward trend in mDFPS across all age groups was observed, with the youngest age group (15–19) experiencing the most significant increase, rising from 21.6% in 2013 to 46.8% in 2022. Women aged 20–35 also show steady growth, reaching 51.6% in 2022, while the 36 + age group experiences more fluctuations, peaking in 2017 at 48.2% before slightly declining. The coverage of mDFPS generally increased with higher levels of education. Women with no formal education started at 30.2% in 2013 and increase to 45.9% in 2022. Those with higher education consistently show the highest mDFPS rates, peaking in 2015 at 58.1% but then declining slightly to 50.4% by 2022. It was further observed that rich women consistently had higher rates of mDFPS than those in the poor category. The wealthiest group saw a rise from 36.2% in 2013 to 49.1% in 2022, while the poorest group increased from 31.6% to 48.8% over the same period. The middle-income group also shows substantial growth, particularly from 2015 onwards. The coverage of mDFPS improved across both urban and rural populations, though rural areas exhibited slightly higher growth in later years. Urban women had an increase from 31.2% in 2013 to 49.2% in 2022, while rural women increased from 34.5% to 50.1% over the same period, suggesting a narrowing of the urban–rural gap in access to modern family planning methods. The coverage of mDFPS increased across countries throughout the years (Figure S1) (See Additional File 2).

### Inequalities in demand for family planning satisfied by modern methods

**Fig. 2:**
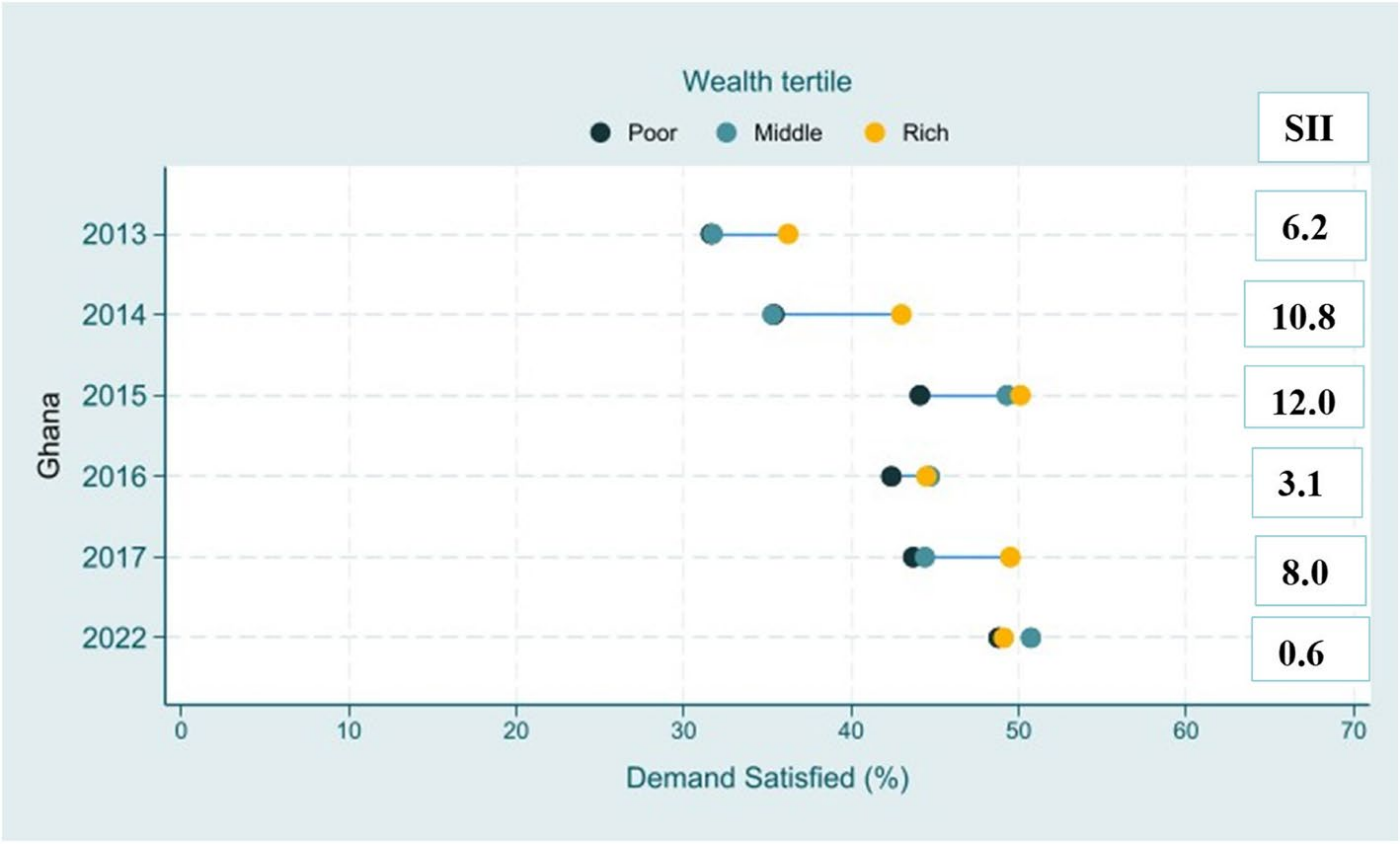
Wealth Inequality Pattern of mDFPS coverage

Inequalities in the coverage of Demand for family planning satisfied by modern methods according to wealth fluctuated over the years (Fig. 2). The coverage was generally higher among wealthier women across the years. However, the SII decreased over the years, suggesting a reduction of disparity in Demand for family planning satisfied by modern methods based on wealth. In 2022, the SII showed that the mDFPS coverage was 0.6 percentage points higher among the wealthiest compared to the poorest indicating a slight positive inequality was observed.

**Fig. 3:**
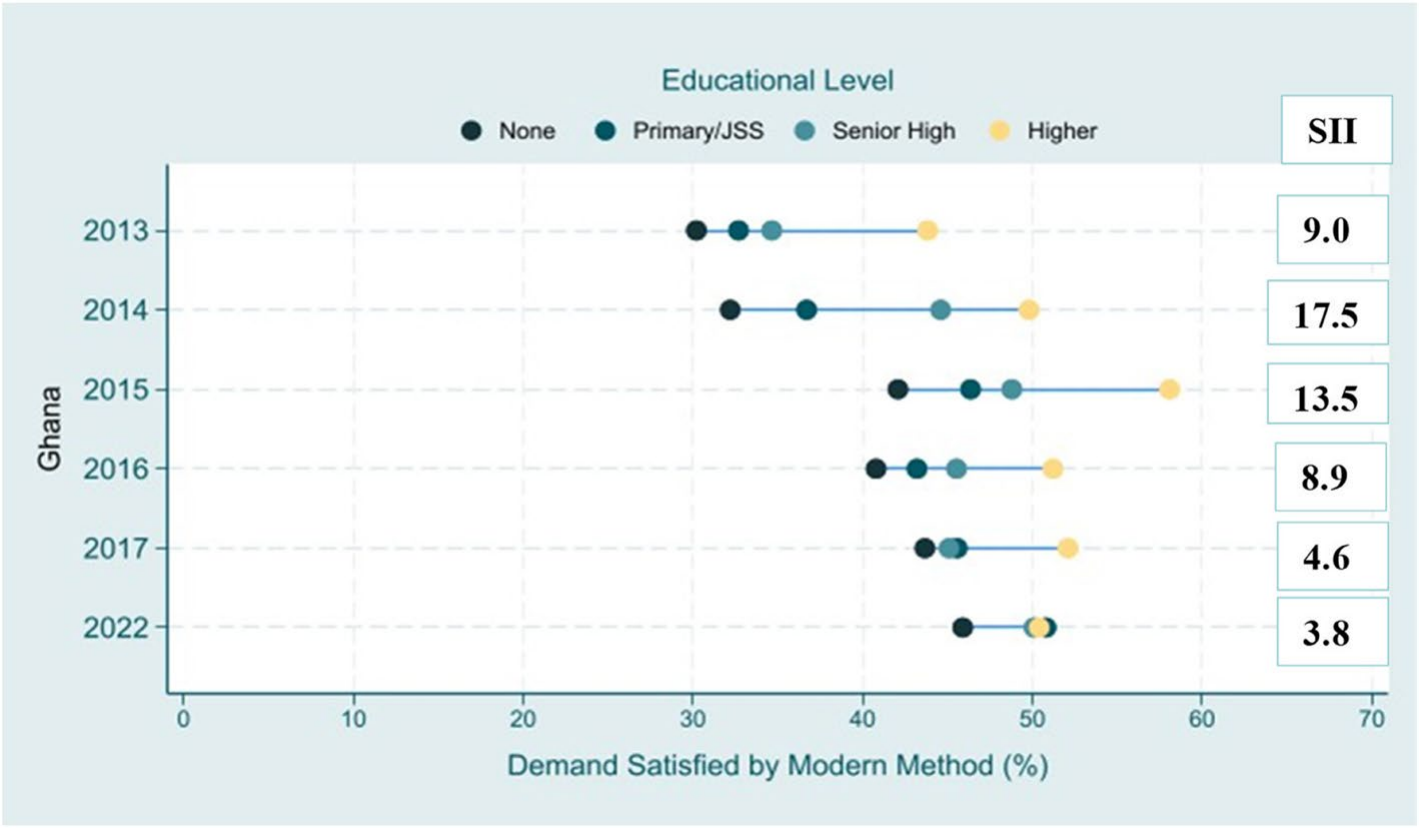
Education Inequality Pattern of mDFPS coverage

On the inequalities according to educational level, women with a higher level of education had a higher coverage of demand for family planning satisfied by modern methods throughout the years (Fig. 3). There was also a notable decrease in the SII for educational level from 2013 to 2014, indicating a reduction of inequality in family planning outcomes based on education. In 2022, a 3.8 percentage point gap was observed between those with no education vs higher education (Table 1).

**Table 1:** SII of mDFPS based on wealth, Educational level, Age and WMADM based on region

Year	Wealth		Educational level		Region
	**SII**	**95%CI**	**SII**	**95%CI**	**WMADM**
**2013**	6.2	−2.9;15.2	9.0	−0.6;18.6	9.4
**2014**	10.8	4.1;17.5	17.5	10.7;24.3	7.7
**2015**	12.0	3.6;20.3	13.5	4.5;22.4	4.8
**2016**	3.1	−6.9;13.3	8.9	−2.2;20.0	3.3
**2017**	8.0	−1.1;17.1	4.6	−5.2;14.41	5.8
**2022**	0.6	−4.6;6.0	3.8	−1.4;9.1	3.5

*SII* Slope of Inequality Index, *WMADM* Weighted Mean Absolute Difference to the Mean

For regional inequalities, a decrease trend in coverage of mDFPS was observed across the years (Figure S1) (See Additional File 2). The northern region had consistent coverage inequalities throughout the years.

### Factors influencing demand for family planning satisfied by modern methods

Age, Region of residence, working status of women and marital status influence the demand for family planning satisfied by modern methods of women aged 15-49 (Table S3) (See Additional File 1). Women aged 20–35 have a 28% increase in odds [95%CI:1.01–1.63; *p* = 0.038] of family planning satisfaction by modern methods compared to those aged 15–19. Mothers currently working have a 27% increased odds of family planning satisfaction by modern methods compared to those who are not working [95%CI: 1.07–1.51; *P* = 0.007]. Furthermore, women who are married or co-habiting have a 33% decrease in odds of family planning satisfaction by modern methods compared to those who are single [95%CI: 0.56–0.84; *P* < 0.000]. Women living in the Ashanti, Brong-Ahafo and Central region have a 56% [95% CI: 1.19–2.05, *p* = 0.001], 27% [95% CI: 1.02–1.59, *p* = 0.031], and 40% [95% CI: 1.08–1.83, *p* = 0.012] increase odds of family planning satisfaction by modern methods compared to those living in the Northern region. Women living in the Eastern, Greater Accra and the Upper East region have a 39% [95% CI: 1.08–1.78, *p* = 0.010], 32% [95% CI: 1.00–1.74, *p* = 0.054], and 93% [95% CI: 1.50–2.49, *p* < 0.001] increase odds of family planning satisfaction by modern methods compared to those living in the Northern region. Furthermore, women living in the Upper West, Volta and the Western region have a 155% [[95% CI: 1.83–3.55, *p* < 0.001], 47% [95% CI: 1.15–1.88, *p* = 0.002] and 64% [95% CI: 1.28–2.10, *p* < 0.001] increase odds of family planning satisfaction by modern methods compared to those living in the Northern region.

## Discussion

In this study, the coverage of women with a demand for family planning satisfied with modern methods increased from 33.0% in 2013 to 49.5% in 2022 with an overall 3.8% annual increase in mDFPS. Coverage from this study is reportedly higher than some African countries including; Chad, Democratic Republic of Congo and Benin in Africa [2] and lower than Tanzania and Ethiopia [28]

The study further revealed that over the years, women who had completed secondary school or tertiary had a greater level of mDFPS in comparison to those who had no education or just completed elementary education. This finding aligns with prior studies and a correlated study conducted in Ghana [20]. Additionally, it was identified that a considerable decrease trend in educational-related inequality in mDFPS coverage from 2014–2022 with women with no education still lagging behind. These decrease trend may be attributed to some initiative implemented by the government at the senior high and tertiary level. The Ghana Education Service (GES)**,** in collaboration with partners such as the United Nations Population Fund (UNFPA) and other NGOs, have introduced Comprehensive Sexuality Education (CSE) in senior high schools which provides age-appropriate information on sexual and reproductive health, covering topics such as family planning, contraception, sexually transmitted infections (STIs), and gender equality [29]. This program is designed to equip young people with the knowledge and skills needed to make informed decisions about their sexual health, reduce teenage pregnancies, and prevent the spread of STIs [30]. Additionally, the Adolescent Health and Development Program (ADHD)**,** led by the Ghana Health Service**,** also targets adolescents and youth in schools and communities [31]. Through these school outreach programs, trained health professionals deliver reproductive health education and counselling, including family planning information, aiming to reduce teenage pregnancies and promote healthy reproductive behaviours among students [31]. Interestingly, in contrast to Ghana, studies from India and Nepal have shown that women with no education have higher mDFPS coverage [32]. This highlights the varying regional dynamics in access to family planning services, suggesting that different strategies may be needed to address disparities across contexts.

Furthermore, this study showed that wealthier women consistently showed higher satisfaction with modern methods levels compared to poorer and middle-income groups across all years. However, a reduction in wealth inequality was observed. This finding is consistent with studies from Ghana and across Africa [2, 20, 32]. This trend implies that efforts to expand access to modern family planning methods in Ghana, including government initiatives, international programs, and partnerships with NGOs, are gradually reaching lower-income populations [33]. Programs including the FP2020 initiatives may have played a role in addressing the financial barriers that have traditionally limited poorer women's access to contraceptives [34]. However, despite the progress, poorer women still face challenges such as limited access to health facilities, lower health literacy, and cultural barriers that may hinder their ability to fully benefit from these services [35].

The Northern region of Ghana has consistently reported lower mDFPS coverage and persistent disparities over the years, despite an overall decline in regional inequalities. Several socioeconomic and cultural factors contribute to this trend, including high unemployment, early marriage, geographical isolation, religious influences, and limited health system capacity [36, 37]. The region’s high poverty rates and lower educational attainment further hinder access to healthcare services, particularly family planning [36, 37]. Additionally, the prevalence of early marriage, polygamy, and high fertility rates negatively correlates with mDFPS utilization [38].

Despite interventions such as the Community-based Health and Planning Services (CHPS) project, aimed at increasing access to family planning services in rural, high-poverty areas, contraceptive prevalence remains low [39]. The Ministry of Health relies heavily on donor support to provide contraceptive commodities across all healthcare levels, ensuring availability even at primary healthcare facilities in the Northern region [39]. However, while community health workers have played a critical role in disseminating family planning information, service delivery through formal healthcare institutions has not significantly shifted attitudes, preferences, or contraceptive use [39]. Strengthening health systems through task-shifting contraceptive counseling to community health workers and enhancing supply chain logistics could help prevent stockouts and improve access. Additionally, community-driven initiatives such as engaging local leaders, promoting male involvement in family planning, and expanding youth-friendly services could be instrumental in overcoming sociocultural barriers and improving mDFPS coverage in the region.

This study found that working women have higher odds of mDFPS compared to those not working. Economic empowerment plays a crucial role, as employed women have greater financial autonomy and decision-making power, which may include the ability to afford and access modern contraceptives [40]. Employment also provides opportunities for greater health literacy, increasing awareness of contraceptive options and reproductive health benefits [41]. Beyond economic empowerment, workplace policies may also contribute to this relationship. Studies indicate that employer-sponsored health insurance, maternity leave, and workplace health programs improve access to family planning counseling and contraception [42]. In settings where workplace policies promote maternal and reproductive health, women may be more likely to utilize modern contraception [41].

In contrast, married or cohabiting women had lower odds of mDFPS compared to those who had never been married. Cultural norms and gender dynamics play a significant role in contraceptive decision-making within marriage or cohabiting relationships. In many societies, male partner influence can strongly shape family planning preferences and contraceptive choices [43]. Some women may prioritize achieving a desired family size before considering contraception, particularly in contexts where having more children is socially or culturally valued [44]. Furthermore, spousal opposition to contraception, lack of joint decision-making, or concerns about fertility-related expectations may discourage the use of modern family planning methods and addressing these barriers through couple-based interventions, male engagement programs, and targeted counseling could improve contraceptive uptake among married women. [44].

### Strengths and limitation

This study leverages large, nationally representative datasets, including the Ghana Performance Monitoring for Action (PMA) and the Demographic and Health Survey (DHS), both known for their methodological rigor and standardized data collection. The diversity of these data sources enhances the study’s ability to capture family planning trends in Ghana. However, reliance on cross-sectional surveys limits causal inference and individual-level trend analysis. Additionally, using multiple data sources introduces potential methodological inconsistencies. To address this, a data harmonization strategy was implemented. Key variables were aligned across surveys to ensure consistent definitions and coding. Sampling differences were adjusted using survey weights from both PMA and DHS. These steps enhance comparability, ensuring more reliable trend estimates and reducing inconsistencies between datasets.

## Conclusion

This study revealed a 3.8% annual average rate change of mDFPS. Although, reductions in both educational and wealth-related inequalities in mDFPS coverage over time, challenges persist in ensuring equitable access, especially for poorer, less educated women and in the Northern region of Ghana. Additionally, working women and those with higher education showed greater odds of mDFPS, highlighting the importance of economic empowerment and educational attainment in reproductive health decision-making. It is recommended that a further district-level inequality analysis be conducted in Ghana to assess the issue at the district level, especially, in the northern region of Ghana.

## Supplementary Information

Supplementary Material 1.

Supplementary Material 2.

## Data Availability

The datasets that were used in the study are publicly available on the DHS website (https://dhsprogram.com/data/available-datasets.cfm?ctryid = 14) and PMA website (https://www.pmadata.org/).

## Abbreviations

DHS: Demographic and health Survey
PMA: Performance action Data
mDFPs: Demand for Family Planning Satisfied by Modern methods
SDGs: Sustainable Development Goals
AARC: Average Annual Rate Change
SII: Slope of Inequality index
WMADM: Weighted Mean Absolute Difference to the Mean
OR: Odds Ratio
AOR: Adjusted Odds Ratio
