# Supplementary material for: Demand for family planning satisfied by modern methods in Ghana: trends and inequalities (2013–2022)
Source: BMC Public Health. 2025 May 1;25:1620. doi: 10.1186/s12889-025-22022-w (PMC12044886; doi:10.1186/s12889-025-22022-w)
Supplement: Supplementary file 1 — Supplementary Material 1. [file 12889_2025_22022_MOESM1_ESM.docx]

**Table S1: Demographic Characteristics of all women of reproductive age (15-49)**

|  | 2013  (n=3606) | 2014  (n=8296) | 2015  (n=5092) | 2016  (n=3689) | 2017  (n=4207) | 2022  (n=15,014) |
| --- | --- | --- | --- | --- | --- | --- |
| Age |  |  |  |  |  |  |
| 15-19 | 18.2 [16.4;20.1] | 18.5 [17.1;20.1] | 20.1 [18.4;21.8] | 18.9 [16.9;21.1] | 19.6 [17.9;21.5] | 17.9 [17.1;18.6] |
| 20-35 | 55.1 [52.8;57.4] | 55.0 [53.2;56.5] | 54.9 [52.8;57.0] | 54.2 [51.8;56.5] | 51.4 [49.2;53.5] | 51.2 [50.0;52.3] |
| 36-49 | 26.7 [25.0;28.4] | 26.5 [25.1;27.9] | 25.0 [23.1;27.1] | 26.9 [25.1;28.8] | 29.0 [27.1;31.0] | 31.0 [30.0;32.0] |
| Educational level |  |  |  |  |  |  |
| None | 21.6 [18.7;24.9] | 20.3 [17.6;23.1] | 18.6 [15.9;21.6] | 16.7 [13.7;20.2] | 16.6 [13.7;20.1] | 15.5 [14.1;16.9] |
| Primary/  JSS | 54.9 [51.7;58.1] | 55.9 [53.1;58.7] | 55.3 [51.3;59.3] | 57.3 [52.5;62.0] | 57.5 [52.9;62.0] | 13.8 [13.3;15.6] |
| Senior  high | 16.6 [14.3;19.1] | 16.8 [15.3;18.3] | 17.5 [15.0;20.3] | 17.7 [15.3;20.4] | 18.1 [16.0;20.5] | 60.5 [58.6;62.3] |
| Higher | 6.9 [5.2;9.0] | 7.1 [5.6;9.0] | 8.6 [6.9;10.7] | 8.27 [4.6;14.3] | 7.7 [4.4;13.1] | 9.7 [8.5;10.9] |
| Residence |  |  |  |  |  |  |
| Urban | 51.1 [47.9-54.3] | 55.9 [51.9;59.8] | 60.1 [53.6;66.2] | 52.5 [45.9;59.0] | 50.2 [44.1;56.4] | 57.0 [55.4;58.6] |
| Rural | 48.9 [45.7;52.1] | 44.2 [40.3;48.1] | 39.9 [33.8;46.4] | 47.5 [41.0;54.1] | 49.8 [43.7;56.0] | 43.0 [41.4;44.7] |
| Region |  |  |  |  |  |  |
| Ashanti | 19.7 [17.2;22.4] | 20.7 [16.6;25.6] | 20.6 [14.1;29.1] | 16.6 [11.2;23.8] | 18.7 [13.7;25.1] | 19.5 [18.0;21.1] |
| Brong-Ahafo | 10.99 [8.32;14.38] | 8.9 [7.3;10.8] | 7.83 [5.7;10.7] | 6.3 [4.8;8.4] | 5.4 [3.9;7.5] | 10.4 [9.7;11.1] |
| Central | 8.57 [7.03;10.41] | 9.8 [7.4;12.9] | 14.3 [8.8;22.3] | 11.0 [7.0;16.8] | 10.2 [7.1;14.1] | 11.3 [10.4;12.4] |
| Eastern | 9.33 [7.60;11.40] | 8.6 [6.8;10.9] | 7.39 [5.0;10.7] | 11.6 [7.6;17.4] | 10.7 [7.6;14.8] | 8.1 [7.5;8.8] |
| Greater Accra | 17.85 [16.17;19.67] | 17.4 [15.4;19.6] | 16.8[13.5;20.8] | 18.7 [14.1;24.2] | 19.5 [14.2;26.2] | 15.5 [14.0;17.1] |
| Northern | 11.46 [9.54;13.71] | 10.0 [8.3;11.9] | 8.94 [5.6;13.9] | 10.0 [7.3;13.5] | 9.9 [7.4;13.3] | 11.7 [10.7;12.8] |
| Upper East | 3.98 [3.32;4.77] | 5.1 [4.4;6.0] | 6.94 [5.6;8.5] | 4.8 [3.3;7.0] | 5.6 [3.3;9.4] | 4.3 [3.6;5.0] |
| Upper West | 2.62 [2.06;3.32] | 4.0 [3.2;4.9] | 4.07 [0.3;5.0] | 3.3 [2.3;4.6] | 3.4 [2.8;4.1] | 2.7 [2.3;3.1] |
| Volta | 6.68 [5.15;8.62] | 5.8 [4.3;7.8] | 4.84 [31.7;7.3] | 5.9 [4.5;7.8] | 7.2 [5.4;9.5] | 7.4 [6.7;8.2] |
| Western | 8.84 [7.51;10.38] | 9.6 [7.2;12.8] | 8.29 [5.9;11.5] | 11.9 [8;17.0.3] | 9.4 [6.8;12.8] | 9.1 [8.4;9.8] |
| Wealth |  |  |  |  |  |  |
| Poor | 33.3 [27.8;39.4] | 33.3 [36.3;47.4] | 33.4 [27.9;39.3] | 33.2 [36.6;50.3] | 33.4 [26.9;40.6] | 33.4 [31.1;35.7] |
| Middle | 33.4 [28.7;38.3] | 33.4 [17.2;24.0] | 33.2 [27.3;39.9] | 33.4 [16.3;23.9] | 33.2 [28.1;38.9] | 33.2 [31.2;35.5] |
| Rich | 33.3 [29.0;38.0] | 33.2 [32.9;43.0] | 33.4 [27.0;40.4] | 33.4 [30.7;43.5] | 33.4 [26.4;41.1] | 33.4 [31.0;35.8] |
| Modern Contraceptive Use | |  |  |  |  |  |
| Yes | 14.10 [12.2;16.2] | 16.5 [14,.6;18.6] | 23.4 [21.0;26.0] | 21.7 [19.3;24.3] | 21.2 [18.0;24.8] | 23.4 [22.4;24.5] |
| No | 85.90 [83.8;87.8] | 83.5 [81.4;85.4] | 76.6 [74.0;79.1] | 78.3 [75.7;80.7] | 78.8 [75.2;82.0] | 76.6 [75.5;77.6] |

**Table S2: Trends in Demand for Family Planning Satisfied by Modern Methods**

|  | 2013  (n=1537) | 2014  (n=3632) | 2015  (n=2427) | 2016  (n=1827) | 2017  (n=1984) | 2022  (n=7020) |
| --- | --- | --- | --- | --- | --- | --- |
| Age |  |  |  |  |  |  |
| 15-19 | 21.6 [14.5;31.0] | 31.6 [22.9;41.8] | 30.9 [24.6;38.0] | 30.0 [22.4;38.9] | 33.6 [27.3;40.6] | 46.8 [42.3;51.4] |
| 20-35 | 34.2 [29.6;39.1] | 38.6 [34.8;42.5] | 50.4 [45.9;54.8] | 47.4 [43.0;51.8] | 46.7 [40.9;52.6] | 51.6 [49.4;53.8] |
| 36+ | 33.9 [27.4;41.0] | 37.6 [31.9;43.8] | 45.9 [38.5;53.4] | 41.1 [33.1;49.6] | 48.2 [41.6;55.0] | 46.5 [43.7;49.4] |
| Educational level | |  |  |  |  |  |
| None | 30.2 [23.8;37.5] | 32.2 [24.4;41.1] | 42.1 [33.0;51.4] | 40.8 [33.0;49.1] | 43.7 [37.2;50.4] | 45.9 [41.9;49.9] |
| Primary  /JSS | 32.7 [28.2;37.5] | 36.7 [32.7;40.9] | 46.4 [41.3;51.6] | 43.2 [39.1;47.4] | 45.6 [40.3;51.0] | 50.8 [47.1;54.6] |
| Senior high | 34.7[26.7;43.6] | 44.6 [39.6;49.7] | 48.8 [41.3;56.3] | 45.5 [37.2;54.1] | 45.1 [35.8;54.8] | 50.1 [48.1;52.1] |
| Higher | 43.8 [30.4;58.3] | 49.8 [39.6;60.1] | 58.1 [48.6;67.0] | 51.2 [40,3;62.0] | 52.1 [34.3;69.4] | 50.4 [45.6;55.3] |
| Wealth |  |  |  |  |  |  |
| Poorest | 31.6 [25.5;38.0] | 35.4 [28.6;42.9] | 44.1 [36.5;48.0] | 42.4 [36.9;48.2] | 43.7 [36.2;51.5] | 48.8 [46.2;51.4] |
| Middle | 31.7 [26.0;38.1] | 35.3 [30.8;40.1] | 49.3 [43.1;55.5] | 44.7 [40.1;49.5] | 44.4 [39.4;49.7] | 50.73 [48.1;53.3] |
| Wealthiest | 36.2 [30.0;43.0] | 43.0 [38.1;48.9] | 50.1 [44.7;55.5] | 44.5 [36.9;52.4] | 49.5 [40.6;58.6] | 49.1 [46.1;52.1] |
| Residence |  |  |  |  |  |  |
| Urban | 31.2 [25.5;37.6] | 36.6 [31.7;41.7] | 48.8 [44.0;53.7] | 45.2 [39.4;51.1] | 46.4 [38.1;54.9] | 49.2 [47.0;51.4] |
| Rural | 34.5 [28.9;40.6] | 38.8 [32.8;45.2] | 44.7 [37.6;52.1] | 42.5 [38.0;47.1] | 44.9 [39.0;51.0] | 50.1 [47.6;52.5] |

**Table S3: Factors Influencing Demand for Family Planning Satisfied by Modern Methods**

| Variable | COR [95%CI] | P-value | AOR [95%CI] | P-value |
| --- | --- | --- | --- | --- |
| Age |  |  |  |  |
| 15-19 | 1.00 |  | 1.00 |  |
| 20-35 | 1.21 [0.99;1.49] | 0.068 | 1.28 [1.01;1.63] | **0.038** |
| 36-49 | 0.99 [0.80;1.22] | 0.911 | 1.00 [0.76;1.35] | 0.949 |
| Educational level |  |  |  |  |
| None | 1.00 |  | 1.00 |  |
| Primary/JSS | 1.22 [0.82;1.26] | 0.063 | 1.06 [0.85;1.33] | 0.616 |
| Senior high | 1.18 [0.77;1.21] | 0.061 | 1.02 [0.82;1.27] | 0.878 |
| Higher | 1.20 [0.74;1.35] | 0.142 | 1.07 [0.80;1.43] | 0.654 |
| Wealth |  |  |  |  |
| Poor | 1.00 |  | 1.00 |  |
| Middle | 1.08 [0.94;1.24] | 0.272 | 1.05 [0.89;1.24] | 0.540 |
| Rich | 1.01 [0.86;1.19] | 0.885 | 1.01 [0.81;1.24] | 0.940 |
| Residence |  |  |  |  |
| Urban | 1.00 |  | 1.00 |  |
| Rural | 1.04 [0.91;1.18] | 0.603 | 1.02 [0.87;1.20] | 0.759 |
| Region |  |  |  |  |
| Northern | 1.00 |  | 1.00 |  |
| Ashanti | 1.72 [1.33;2.21] | **<0.001** | 1.56 [1.19;2.05] | **0.001** |
| Brong Ahafo | 1.34 [1.09;1.66] | 0.006 | 1.27 [1.02;1.59] | 0.031 |
| Central | 1.56 [1.22;2.00] | **0.001** | 1.40 [1.08;1.83] | **0.012** |
| Eastern | 1.53 [1.21;1.94] | **<0.001** | 1.39 [1.08;1.78] | **0.010** |
| Greater Accra | 1.36 [1.06;1.76] | **0.017** | 1.32 [1.00;1.74] | **0.054** |
| Upper East | 1.95 [1.55;2.47] | **<0.001** | 1.93 [1.50;2.49] | **<0.001** |
| Upper West | 2.49 [1.80;3.45] | **<0.001** | 2.55 [1.83;3.55] | **<0.001** |
| Volta | 1.54 [1.22;1.96] | **<0.001** | 1.47 [1.15;1.88] | **0.002** |
| Western | 1.80 [1.43;2.26] | **<0.001** | 1.64 [1.28;2.10] | **<0.001** |
| Number of living Children | 1.03 [0.99;1.08] | 0.515 | 1.03 [0.99;1.08] | 0.176 |
| Women currently Working |  |  |  |  |
| No | 1.00 |  | 1.00 |  |
| Yes | 1.25 [1.06;1.47] | **0.007** | 1.27 [1.07;1.51] | **0.007** |
| Marital status |  |  |  |  |
| Never Married | 1.00 |  | 1.00 |  |
| Married/Living with a partner | 0.75 [0.64;0.87] | **<0.001** | 0.67 [0.56;0.84] | **<0.001** |
| Widowed/separated/Divorced | 1.45 [1.09;1.91] | **0.010** | 1.29 [0.96;1.74] | 0.091 |

COR= Crude odds ratio; AOR= Adjusted odds ratio; 95%CI= 95% Confidence interval
