# Supplementary material for: Demand for family planning satisfied by modern methods in Ghana: trends and inequalities (2013–2022)
Source: BMC Public Health. 2025 May 1;25:1620. doi: 10.1186/s12889-025-22022-w (PMC12044886; doi:10.1186/s12889-025-22022-w)
Supplement: Supplementary file 2 — Supplementary Material 2. [file 12889_2025_22022_MOESM2_ESM.docx]

***Figure S1: Map Showing Regional Trends on Coverage of mDFPS. [A=2013; B=2014; C=2015; D=2016, E=2017; F=2022]***

***Figure S2: Regional Inequality Pattern of mDFPS.*** [Horizontal lines represent national coverage, Blu bars represent regional coverages, Red bars represent the Weighted Mean Absolute Difference to the Mean].
